# Low glomerular filtration rate values are associated with higher TSH in an elderly population at high cardiovascular disease risk

**DOI:** 10.3389/fendo.2023.1162626

**Published:** 2023-08-16

**Authors:** Gabriela Brenta, Alejandra Nepote, Adriana Barreto, Carla Musso, Cristina Faingold, Pía Fossati, Alessandro Antonelli, Poupak Fallahi, Fausto Famá, Tomás Meroño

**Affiliations:** ^1^ Endocrine Division, Unidad Asistencial Dr Cesar Milstein, Buenos Aires, Argentina; ^2^ Department of Clinical and Experimental Medicine, University of Pisa, Pisa, Italy; ^3^ Department of Human Pathology in Adulthood and Childhood “G. Barresi”, University of Messina, Messina, Italy; ^4^ Faculty of Pharmacy and Food Sciences, University of Barcelona, Barcelona, Spain

**Keywords:** older adults, subclinical hypothyroidism, kidney failure, cardiovascular risk, renal failure

## Abstract

**Background:**

Hypothyroidism is associated with impaired glomerular filtration rate (GFR), a recognized cardiovascular disease (CVD), and mortality risk factor. In older adults, this association remains unexplored. We aimed to determine the relationship of elevated TSH with GFR in an elderly population at high CVD risk.

**Methods:**

Older adults (age>65ys) with high CVD risk defined by two or more CVD risk factors: smoking (S), high blood pressure (HBP), high total cholesterol, low HDL cholesterol, diabetes (DM), metabolic syndrome or previous cardiovascular event, were prospectively included at our ambulatory Endocrine Clinic. Patients under levothyroxine or thyroid disease were excluded. TSH> 6mU/l defined subclinical hypothyroidism (ScH) with normal free T4 levels. Estimated GFR was calculated by the Berlin-Initiative Study (BIS)-1 formula for elderly population. Urinary albumin to creatinine ratio (uACR), IL-6 and TNF-α, and Carotid intima-media thickness (CIMT) were also determined. The U Mann-Whitney test, the Spearman test, and multiple linear regression were used as statistical tests,

**Results:**

Finally 246 patients (68% females) were included and 20 (8%) had ScH. This group, was older (median, Q1-Q3: 77,72-78; 72,68-77 years, p=0.01) and DM was less frequent than in the euthyroid group (35 vs 58%, p=0.039). Lower fasting glucose (-20%,p=0.01), GFR (-14%,p=0.01) and freeT4 (-10%,p<0.001) were found compared to euthyroid patients. A higher prevalence of Kidney failure was found in ScH (80 *vs.* 46%, p=0.003) vs. euthyroid individuals. Significant correlations with GFR were detected: age (r-0.482,p<0.001), TSH (r-0.172,p=0.004), IL-6 (r-0.150,p=0.047), TNF-α (r-0.274,p<0.001), uACR (r-0.170,p=0.009) and CIMT(r-0.189,p=0.004). By multiple linear regression, in a model adjusted by age, sex, BMI, uACR, S, DM, TNF-α and HBP, TSH (Bst -0.14, p=0.023, R^2^ = 0.25) was found an independent predictor of GFR.

**Conclusion:**

In older adults with high CVD risk, ScH is associated with lower renal function, and this relationship is present regardless of other cardiometabolic risk factors. These results suggest that ScH could contribute to low GFR and excess CVD risk, although this hypothesis should be addressed in longitudinal studies.

## Introduction

The relationship between the thyroid and the heart has been extensively reviewed illustrating all the plausible mechanisms that can link low thyroid function to cardiovascular risk (1). Furthermore, research in recent years has demonstrated that overt and subclinical hypothyroidism are associated with higher mortality rates in the general population (2–4). By contrast, findings in older adults are conflicting. Although increased all-cause mortality has been reported in overt hypothyroid elderly patients (5, 6), subclinical hypothyroidism (ScH) is generally considered without consequences. This is mainly because TSH levels are naturally higher with advanced age (7) and, most notably, due to the lack of evidence of a significant impact on mortality rates above 65 years old (8). Furthermore, there is no clear benefit on cardiovascular outcomes (8) or overall mortality (9) upon levothyroxine replacement in patients with advanced age. However, it is still not clear if ScH may have a detrimental effect on specific subgroups of older adults, such as those with preexistent cardiovascular disease (CVD) risk.

It has long been known that thyroid hormones regulate renal homeostasis. Thyroid hormones have been involved in determining kidney size and structure during development and renal function by their action on cardiac output, intra-renal hemodynamics, renin-angiotensin-aldosterone production, and glomerular-tubular feedback (9).

A growing body of evidence suggests that renal function deteriorates in overt hypothyroid patients (10). It is of particular interest that a higher prevalence of hypothyroidism has been reported in patients with reduced glomerular filtration rate (GFR) and those in dialysis (11).

Although controversy still exists (12), according to a recent meta-analysis, ScH is significantly associated with a higher risk of Chronic Kidney Disease (CKD). Still, this association was mainly observed in those subjects younger than 70 years (13).

It should be stated that end-stage renal disease patients have a higher mortality risk compared to the general population (14). The leading causes of CKD are hypertension, obesity, and diabetes mellitus, and even with appropriate treatment, the progression of CKD and its associated cardiovascular risk cannot be effectively prevented (15).

Given that CKD is considered a risk factor for CVD and is more frequently observed at an advanced age, a mild decrease in thyroid hormones may negatively impact the renal function of older adults with ScH. This study aimed to assess the association between thyroid and renal function in older adults with high CVD risk.

## Subjects and methods

### Subject study

We prospectively included in a consecutive way patients at our ambulatory care center of Endocrinology that were ≥ 65 years old and had high CVD risk. None had been admitted to a hospital during the last six months. The ethics committee approved the protocol and all patients signed informed consent to participate in this cross-sectional study. In 1 year, 246 patients were included out of 309 eligible individuals.

Two or more CVD risk were required to define high CVD risk factors. Among the CVD risk factors were: high blood pressure (HBP), smoking (S), diabetes (DM), high total cholesterol >200mg/dl, low HDL cholesterol <40mg/dl, previous cardiovascular event, metabolic syndrome (MetS).

MetS was defined by three of the five following criteria according to the harmonized IDF criteria (16): waist circumference (WC) (for European population) ≥ 94 cm for men, and ≥ 80 cm for women, elevated triglycerides (TGs) ≥ 150 mg/dL, or drug treatment for high TGs, reduced serum high-density lipoprotein cholesterol (HDL-C) < 50 mg/dL for men and < 40 mg/dL for women or drug treatment for reduced HDL-C, elevated blood pressure [systolic (SBP) ≥130 and diastolic (DBP) ≥85 mmHg or antihypertensive drug treatment in a patient with a history of hypertension, and elevated fasting glucose ≥ 100 mg/dL or drug treatment for high glucose as an alternate indicator.

Those patients with known thyroid disease and L-T4 use were excluded.

### Subject interview

Two certified endocrinologists interviewed face-to-face all eligible patients. They were asked to respond about their personal and family history of diseases regarding high blood pressure, CVD, thyroid disease, dyslipidemia and DM. They also responded about smoking habits, physical activity, medication, and diet. The definition of DM was based on the patient´s self-report, the clinical record, or the intake of antidiabetic drugs.

### Clinical and anthropometric assessment

Blood pressure was the average of a triple assessment in both arms with a sphygmomanometer in a sitting position. To measure height and weight, we used a manual scale. To measure waist circumference (WC), we used a folding tape at the natural waistline. To calculate Body mass index (BMI), we divided weight (kg) by the square of height (cm).

### Biochemical evaluation

Samples of venous blood were obtained early in the morning in fasting conditions. To evaluate thyroid function, we measured thyrotropin (thyroid-stimulating hormone, TSH), free thyroxine (free T4), triiodothyronine (T3), and the anti-thyroid antibody peroxidase (TPOAb). Immunochemiluminescent assay was employed with an automatic analyzer (IMMULITE 1000, Siemens). The range values for TSH levels were 0.3–6 mU/L, for free T4 0.6-1.8 ng/dL, and for T3: 80–190 ng/dL. The Inter-assay coefficients of variation for TSH were 5.7–8.3%, for free T4 5.7–10.6%, and for T3, 5.6–11.6%.

TSH>6mU/l with normal free T4 levels was considered subclinical hypothyroidism (ScH).

The metabolic parameters measured were total cholesterol (RV normal < 200, borderline 200– 239, high > 239mg/dL) with an enzymatic colorimetric assay (CV 2.5%); low-density lipoprotein cholesterol (LDL-C) (RV, normal < 100, borderline 100–129, intermediate 130–159, high > 160mg/dL) with the Friedwald formula or homogeneous assay by (Cobas 311, Roche) (CV 2%), HDL-C [RV, male, with low cardiovascular risk (CVR) > 55, moderate CVR 35–55, high CVR < 35; female, low CVR > 65, moderate CVR 45–65, high CVR < 45 mg/dL] with an homogeneous colorimetric assay (CV 2.5%); TGs (RV 40- 150 mg/dL) with an enzymatic colorimetric assay (CV 2%); glycated hemoglobin (HbA1c) (RV 4.8–5.9%), with immunoturbidimetric assay (Cobas 311, Roche) and glycemia (range values [RV] 70–110mg/dL), with an enzymatic colorimetric assay (Cobas 311, Roche);

The renal function assessment included the albuminuria assay with a morning urine sample analyzed by immunochemiluminescent assay IMMULITE 1000 Siemens (CV 8.3%; VR to 30mg albumin/gram creatinine). Urinary albumin to creatinine ratio was calculated (uACR).

Estimated (e)GFR was estimated with a formula developed for older adults, the Berlin-Initiative Study (BIS)-1 formula (17). The Berlin-Initiative Study (BIS)-1 formula:

3736 x creatinine - 0.87 x age - 0.95 x 0.82 (if female). (Creatinine in mg/dl).

Stages of chronic kidney disease were defined based on eGFR according to Kidney failure: Improving Global Outcomes (KDIGO) (18).

Kidney failure was characterized as eGFR<60ml/min or uACR>30mg/g.

Proinflammatory markers IL-6 y TNF-α were evaluated by ELISA (R&D Systems, USA).

### Noninvasive examination of the walls of peripheral arteries

The assessment of carotid intima-media thickness (CIMT) was determined by B-mode ultrasonographic imaging (probe 7.5 MHz, Siemens, EEUU) of the carotid arterial walls. The distance between the arrows from the lumen–intima interface to the media–adventitia interface was considered for CIMT measurement. Only two operators performed the evaluations in all cases. A 7.5 MHz probe was used according to international recommendations (19).

### Sample size calculation

Sample size was calculated considering previous studies showing a correlation coefficient between TSH and eGFR ranging (-0.20 and -0.30) (20, 21). Therefore, with an effect size of 0.2, an alfa error of 0.05 and a power of 0.80 the required sample size was 191 participants. G*power (University of Dusseldorf, Germany) software was used for sample size calculations.

### Statistical analysis

Results are expressed as median and inter-quartile range. The U Mann-Whitney test was used to establish differences among results, and correlations were obtained using the Spearman test. Logistic regression was used to establish the association between Kidney disease and ScH, adjusted by age. Multivariable linear regression was used to identify independent predictors of eGFR. We tested two models, the first model was adjusted only for age and sex, and the second model was adjusted for the variables that showed a statistically significant correlation with eGFR.

A *p-value* < 0.05 was considered statistically significant. SPSS 25.0 (IBM, USA) was used for statistical analyses.

## Results

### Characteristics of the whole population

**Figure 1:**
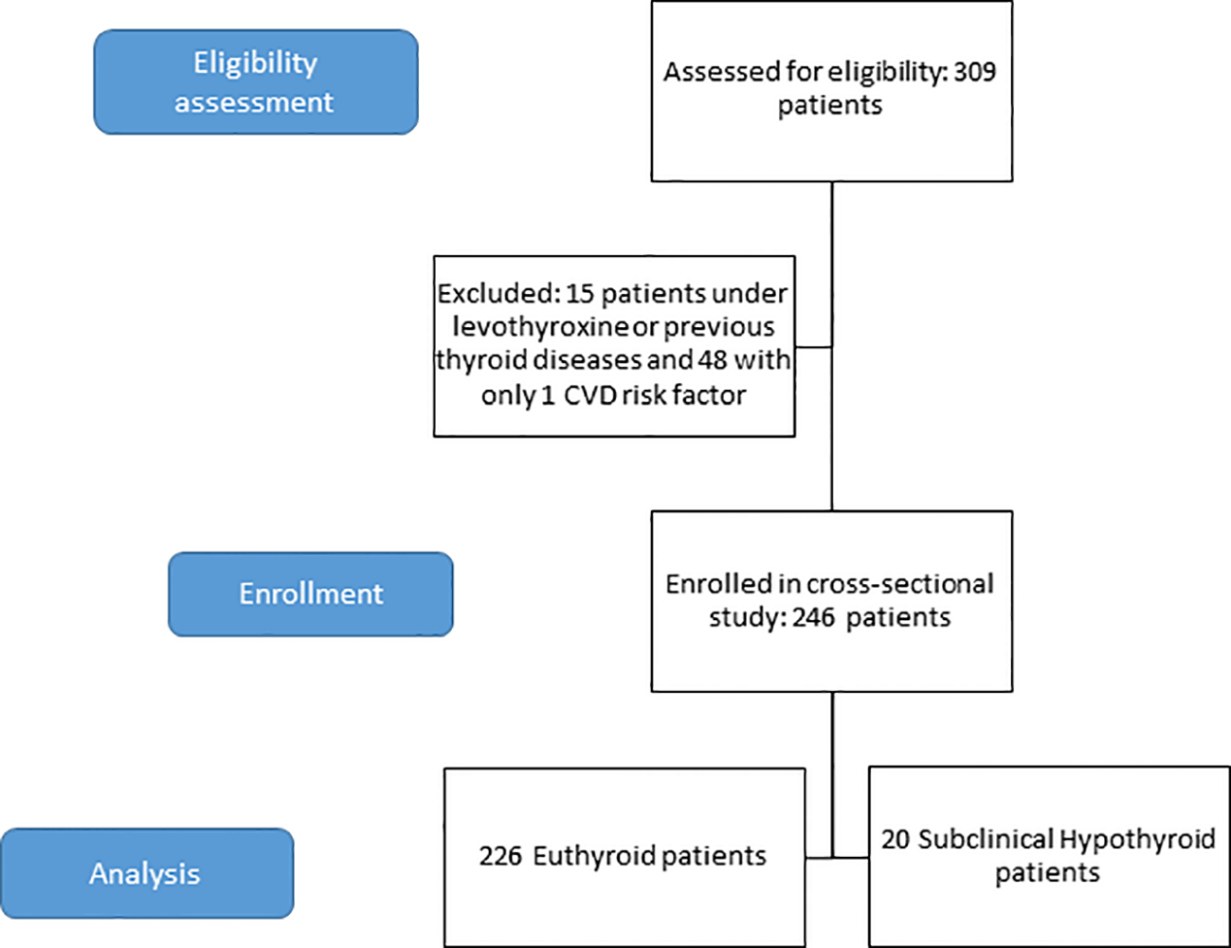
Flow diagram of the included population.

This cross-sectional study finally included 246 patients (68% female sex) aged (median, Q1-Q3) 73 (68–77), years (**Figure 1** and **Table 1**).

**Table 1:** Characteristics of the population, subdivided into ScH and euthyroid groups.

	Total (n:246)	Euthyroidism (n:226)	ScH (n:20)
AGE years (Median (q1-q3))	73 (68–77)	72 (68-77)	77 (72-78)*
Female sex n(%)	167 (68)	149 (66)	17 (85)
Central obesity n(%)	206 (85)	190 (85)	16 (80)
HBP or in treatment n(%)	226 (92)	207 (91)	19 (95)
Low HDL-C n(%)	83 (34)	78 (34)	5 (25)
Hypertriglyceridemia n(%)	92 (37)	84 (37)	8 (40)
IFG n(%)	194 (79)	184 (81)	10 (50)*
MetS n(%)	165 (75)	173 (76)	12 (60)
DM n(%)	140 (57)	133 (59)	7 (35)*
CIMT mm. (Median (q1-q3))	0.85 (0.75-0.95)	0.85 (0.75-0.95)	0.85 (0.75-.95)
TSH mU/L (Median (q1-q3))	2.5 (1.6-3.8)	2.4 (1.4-3.5)	7.1 (6.5-9.3)*
FT4 ng/dL (Median (q1-q3))	1.1 (1-1.2)	1.1 (1-1.2)	1 (0.9-1.1)*
T3 ng/dL (Median (q1-q3))	115 (101-130)	115 (101-130)	109 (100-132)
+TPOab n(%)	37 (15)	32 (14)	5 (25)
uACR	10 (5.7-23)	10 (5.6-23)	9.3 (6.1-19)
uACR>30 n(%)	48 (20)	45 (20)	3 (16)
Kidney failure n(%)	119 (48)	103 (46)	16 (80)*

ScH, subclinical hypothyroidism; HBP, high blood pressure; HDL, High-density lipoprotein; IFG, Impaired fasting glucose; MetS, Metabolic Syndrome; DM, Diabetes Mellitus; CIMT, Carotid intima-media thickness; TSH, Thyrotropin; FT4, free Thyroxine; T3, Triiodothyronine; +TPOab, positive thyroperoxidase antibody; uACR, urinary albumin-creatinine ratio.

*p<0.05 vs. Euthyroidism.

The information on cardiovascular risk factors showed that 28% of the patients were former smokers, 6% were alcoholics, 67% had dyslipidemia, 79% had HBP, 56% had DM, and 17% had a history of CVD.

Regarding hereditary factors, 52% had a family history of DM, 58.5% had HBP, 39% had CVD, 25% had dyslipidemia, and 18.6% had thyroid disease.

When patients were interviewed about the treatment they were receiving, it was shown that 35.6% performed physical activity, 39.3% were on a diet, 41.1% on metformin, 16.9% on a sulphonylurea, 2% on glinides, 1.2% on thiazolidinedione, 7.4% on insulin, 6.6% on incretins, 0.4% Sodium-glucose Cotransporter-2 (SGLT2) Inhibitors, 2.4% on acarbose, 35.3% on Angiotensin-converting enzyme (ACE) inhibitors, 37.8% on Angiotensin II receptor antagonists (ARA-II), 30.3% on beta-blockers, 17.4% on calcium blockers, 19.1% on diuretics, 41.7% on statins, 11.2% on fibrates and 2.8% on nicotinic acid.

### Subclinical hypothyroidism

The prevalence of ScH was 8% (n: 20) and 25% of these patients were TPOab positive. It was shown that ScH was more prevalent in female than male patients, 10.1 vs. 3.7%, although the difference did not attain significance (*p*=0.06)

Despite an older age in the ScH group of patients (median, inter-quartile range): 77, 72-78 *vs.* 72, 68-77 years; p=0.01), the prevalence of DM was lower in this group (35 *vs.* 58%, p=0.039).

In addition, patients with ScH had lower levels of glycemia (median, inter-quartile range): 101, 89-134 vs 121, 103-141 mg/dL (p=0.01), GFR 57.4, 47-64.7 vs 64.6, 56.7-73.6 ml/min. (p=0.01) and free T4 levels 1, 0.9-1.1 vs 1.1, 1-1.2 ng/dL (p<0.001) (**Table 1**).

### Renal function and CKD

Twenty percent of patients had increased uACR levels in the whole group, and 36.9% had low eGFR. The KDIGO staging of all patients showed: Stage 1: 6%; Stage 2: 56%; Stage 3a: 30%; Stage 3b: 8%. None of the patients had an eGFR<30ml/min.

**Figure 2:**
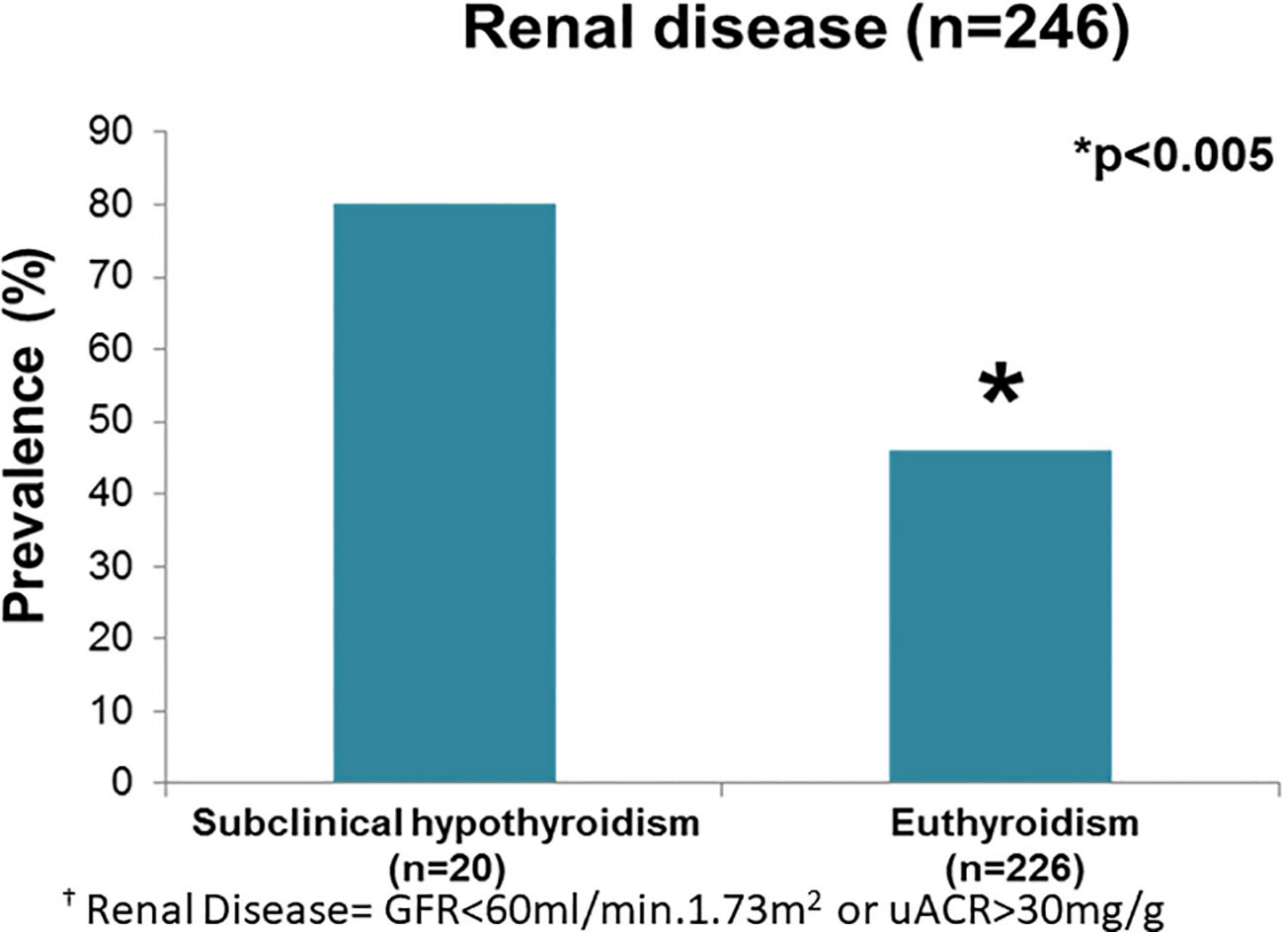
Prevalence of renal disease comparing patients with Subclinical Hypothyroidism with Euthyroid patients.

ScH patients had a higher prevalence of Kidney failure (80 *vs.* 46%, p=0.003) than euthyroid individuals. (**Figure 2**). The association of Kidney failure and ScH was still present after age adjustment (odds ratio (OR) 2.98, 95% CI 1.08-8.73, *p*=0.046).

In those patients with Kidney failure, ScH was more prevalent than in those with preserved renal function (13 vs 3.8%, *p*=0.008).

Given that female sex is associated mainly with the presence of autoimmune thyroid disease, the analysis was further divided according to sex, and it was revealed that women with Kidney failure had higher TSH values (median, Q1-Q3) 3.2, 2.3-5.2 vs. 2.7, 1.5-3.8 mU/L, *p*=0.006) and a more considerable prevalence of ScH (17.5% vs 3.4%, *p*=0.03) than those with preserved renal function. In men, these differences, however, were not observed.

### eGFR association with TSH values considering the presence of other anthropometric, biochemical, and ultrasound parameters

eGFR correlated with age, TSH, IL-6, TNF-α, uACR, and CIMT (**Table 2**).

**Table 2:** Correlations between eGFR and TSH, age, TNF-α, IL-6, CIMT and uACR.

eGFR	r	*p*
Age	-0.48	<0.001
TSH	-0.17	0.004
TNF-α	-0.28	<0.001
IL-6	-0.150	0.047
CIMT	-0.18	0.004
uACR	-0.17	0.004

eGF, Estimated Glomerular Filtration Rate; TSH, Thyrotropin; TNF-α, Tumor Necrosis alpha; IL-6, Interleukin 6; CIMT, Carotid intima-media thickness; uACR, urinary albumin-creatinine ratio.

In the multiple regression analysis, TSH resulted as an independent predictor of eGFR in a model adjusted by age, sex, BMI, uACR, TNF-α, smoking, DM, and HBP (Bst -0.14, p=0.023, R^2^ = 0.25) (**Table 3**).

**Table 3:** Multivariable linear regression used to identify independent predictors of eGFR.

Model	Variable	Beta_st_	t-value	p-value	95% CI for B		R^2^
Model 1	LogTSH	-0.13	-2.32	0.021	-12.8	-1.05	0.24
	Age	-0.48	-8.49	<0.001	-1.4	-0.8	
	Sex	0.02	0.41	0.68	-2.7	4.1	
Model 2	LogTSH	-0.14	-2.29	0.023	-14.803	-1.119	0.25
	Age	-0.41	-6.44	<0.001	-1.325	-0.703	
	Sex	0.01	0.20	0.837	-3.509	4.330	
	BMI	0.15	2.43	0.016	0.078	0.748	
	uACR	-0.15	-2.51	0.013	-9.307	-1.114	
	HBP	-0.13	-2.07	0.04	-8,662	-0.207	
	DM	0.13	2.06	0.041	0.157	7.205	
	Smoking	-0.06	-1.05	0.29	-5.729	1.749	
	TNF-α	-0.19	-3.13	0.002	-0.888	-0.201	

eGF, Estimated Glomerular Filtration Rate; TSH, Thyrotropin; BMI, Body Mass Index; HBP, High Blood Pressure; DM, Diabetes Mellitus; TNF-α, Tumor Necrosis alpha; uACR, urinary albumin-creatinine ratio.

## Discussion

This study shows the cross-sectional association between ScH and CKD in older adults with high CVD risk. Lower renal function was observed in the ScH group compared to the euthyroid group of patients. TSH levels were significantly negatively associated with eGFR after adjusting for several confounders such as age and sex, BMI, uACR, TNF-α, smoking, DM, and HBP.

The association between nephropathy and lower thyroid function has been well depicted by a series of overt hypothyroid patients in whom kidney dysfunction was detected (22). Subclinical hypothyroidism has also been related to CKD. This is particularly evident in DM patients with ScH, in whom diabetic complications are more prevalent than in euthyroid patients (23). According to a meta-analysis by Han et al. (24), an overall OR of 1.74 (95% CI, 1.34-2.28) for diabetic nephropathy was shown in ScH vs. euthyroid patients. This meta-analysis assessed ten cross-sectional studies with 5,768 T2DM patients and 6,225 nondiabetic patients. Moreover, in a cohort study of 5936 T2DM in whom 362 patients with ScH were included, it was described that eGFR was significantly lower, uACR levels higher, and the incidence of CKD was increased in the ScH group when compared to the rest of the population (25). These reports align with what was found in this study of older adults with high CVD risk. Most of them had DM or MetS, and about one-third had low renal function despite receiving adequate antihypertensive or antidiabetic treatment. Regarding thyroid dysfunction, the proportion of ScH in the whole population was similar to what has been described for general population worldwide (24), although it was significantly higher in those patients with kidney disease. Intriguingly, the frequency of DM was lower than in the euthyroid group of patients. Nonetheless, ScH patients had lower eGFR suggesting that this finding can be independent of DM and its microangiopathic complications. Furthermore, the negative association between TSH with eGFR remained even with adjustment for DM.

Another controversial issue is if hypothyroidism can trigger renal dysfunction or if it is instead its consequence. In this line, prospective longitudinal studies might help to answer this question. As opposed to what has been observed in cross-sectional studies, in the Atherosclerosis Risk in Communities (ARIC) study, the development of incident CKD in middle-aged adults was not related to impaired thyroid function (26). Similar conclusions were obtained in a meta-analysis including longitudinal studies from 16 different cohorts performed in the general population (27). Although there was a clear initial relationship between hypothyroidism and lower renal function, low thyroid function was not associated with a long-run deterioration of renal function. A possible interpretation of these findings may be reverse causality, renal dysfunction being the cause of thyroid hormone alterations. However, a recent Mendelian randomization study proved the opposite (28), supporting the idea that hypothyroidism, increased TSH, and TPOAb may cause decreased eGFR and increased CKD. In support of the latter theory, in a prospective study with a follow-up period of 26.1 months, ScH was associated with worse renal function when compared to euthyroid individuals (29).

The clinical relevance and prognostic significance of ScH in CKD patients has been demonstrated by the Transition of Care in CKD (TC-CKD) study (30). In this large cohort of 15 335 patients it was shown that TSH levels >5.0 mIU/L were related to mortality in end-stage renal disease patients. In this connection, another support towards a potential adverse effect of ScH in CKD would be a response to levothyroxine treatment. Although there is no consensus on the role of levothyroxine treatment in ScH, there is some partial evidence that ScH patients with diabetic nephropathy might benefit from treatment (31, 32). No large randomized controlled trial has been conducted to assess the benefit of levothyroxine in CKD + ScH, so the clinical relevance of thyroid hormone replacement in this setting is yet to be explored. Some of the available data have been obtained in a retrospective observational cohort study of US Veterans, where levothyroxine-treated (n: 157) and nontreated CKD (n:157) + ScH patients followed for 24 months were analyzed for clinical outcomes. Despite an absence of overall benefit on eGFR or CKD progression, it was revealed that levothyroxine use was associated with a lower length of hospital stay for reasons related to CKD, which may translate into a possible economic benefit (33). Furthermore, in a previous retrospective Korean study, the decrease in renal function in 113 CKD patients with ScH was attenuated with levothyroxine after two years (34). With all this background, our findings of reduced GFR in patients with higher TSH values suggest that ScH may not represent a mere bystander and could be, in part, the culprit of the deterioration of renal function in older patients.

One of the strengths of this study is its focus on an advanced age segment of the population, most vulnerable to CKD consequences. Most recently, in a retrospective study of 285 hypothyroid older adults, it was shown that eGFR improved upon levothyroxine treatment (35). However, one of the most extensive studies on older adults was performed in Australia and included 1571 community-dwelling older adults, out of which 125 had thyroid dysfunction. In that group, eGFR < 60 ml/min/1.73 m2 was more prevalent than in euthyroid individuals (36). Those patients in the higher quartile of TSH had 82% increased odds of having CKD than those in the first quartile. The HUNT study provided evidence that low thyroid function, even within the clinically normal range, may be associated with CKD in older adults (20). In patients over 40 years of age, an increase of TSH levels even within the reference range was negatively associated with eGFR. In another cohort study of older Taiwanese adults, hypothyroidism and CKD development were associated even in the absence of DM (37). Similarly, in another study of 378,101 patients more than 55 years old, those with hypothyroidism were 25% more prone to have CKD than those who were euthyroid (38).

Compared to all these studies performed on older adults, our population is much smaller, yet it reproduces similar findings in a specific set of high CVD risk subjects. Most importantly, although most individuals had MetS, DM, or hypertension, they were not undergoing any critical illness that may influence their interpretation of the thyroid profile.

Although we could not assess outcomes due to the study’s cross-sectional design, lower eGFR levels were correlated to higher CIMT and inflammatory marker levels, which supports the concept of a higher CVD risk in patients with lower renal function. ScH screening is advocated in the older adult population, but its management is incredibly controversial. These findings confirm the need for screening older adults with TSH measurements (39) and point towards a specific group with higher CVD risk who may eventually benefit from levothyroxine treatment.

This study’s limitations are given by its cross-sectional design, which does not allow for clarifying a causal association between lower renal function and hypothyroidism and the definition of ScH with a single TSH measurement. Higher TSH values in the presence of lower T3 values could be interpreted as a recovery phase of NTI in patients who had been recently admitted for an acute illness (40). Still, it was not the case in this series of patients recruited at an ambulatory care center. Furthermore, the proportion of patients with low T3 levels was negligible.

It is in fact known that hemoglobin levels are positively related to GFR, particularly among aged persons (41, 42). Anemia is a known clinical sign associated with hypothyroidism. Thyroid hormones stimulate the proliferation of erythrocytes, both directly and indirectly through increased production of erythropoietin, while sideropenic anemia negatively affects thyroid hormone status, since iron is vital for the activity of thyroid peroxidase (43). Therefore, another limitation of this study is the lack of hemoglobin levels evaluation.

## Conclusion

Increased TSH serum values in older adults with high CVD risk are associated with lower renal function. This association is independent of traditional CVD risk factors, including diabetes and hypertension. Given that CKD is a major health problem, its link with lower thyroid function deserves full recognition.

## Data availability statement

The raw data supporting the conclusions of this article will be made available by the authors, without undue reservation.

## Ethics statement

The requirement of ethical approval was waived for the studies involving humans. The studies were conducted in accordance with the local legislation and institutional requirements. Written informed consent for participation was not required from the participants or the participants’ legal guardians/next of kin. Written informed consent was obtained from the individual(s) for the publication of any potentially identifiable images or data included in this article.

## Author contributions

GB, TM, CF, and CM contributed to the conception and design of the study. PFo, AB, and AN organized the database. TM performed the statistical analysis. GB wrote the first draft of the manuscript. AA, PFa, FF, and GB wrote sections of the manuscript. All authors contributed to manuscript revision, read, and approved the submitted version.
